# The Combination of Hydrogels with 3D Fibrous Scaffolds Based on Electrospinning and Meltblown Technology

**DOI:** 10.3390/bioengineering9110660

**Published:** 2022-11-07

**Authors:** Jakub Erben, Radek Jirkovec, Tomas Kalous, Marketa Klicova, Jiri Chvojka

**Affiliations:** Department of Nonwovens and Nanofibrous Materials, Faculty of Textile Engineering, Technical University of Liberec, 461 17 Liberec, Czech Republic

**Keywords:** electrospinning, meltblown, bioprinting, scaffold, hydrogels, nanofibers

## Abstract

This study presents the advantages of combining three-dimensional biodegradable scaffolds with the injection bioprinting of hydrogels. This combination takes advantage of the synergic effect of the properties of the various components, namely the very favorable mechanical and structural properties of fiber scaffolds fabricated from polycaprolactone and the targeted injection of a hydrogel cell suspension with a high degree of hydrophilicity. These properties exert a very positive impact in terms of promoting inner cell proliferation and the ability to create compact tissue. The scaffolds were composed of a mixture of microfibers produced via meltblown technology that ensured both an optimal three-dimensional porous structure and sufficient mechanical properties, and electrospun nanofibers that allowed for good cell adhesion. The scaffolds were suitable for combination with injection bioprinting thanks to their mechanical properties, i.e., only one nanofibrous scaffold became deformed during the injection process. A computer numerical-control manipulator featuring a heated printhead that allowed for the exact dosing of the hydrogel cell suspension into the scaffolds was used for the injection bioprinting. The hyaluronan hydrogel created a favorable hydrophilic ambiance following the filling of the fiber structure. Preliminary in vitro testing proved the high potential of this combination with respect to the field of bone tissue engineering. The ideal structural and mechanical properties of the tested material allowed osteoblasts to proliferate into the inner structure of the sample. Further, the tests demonstrated the significant contribution of printed hydrogel-cell suspension to the cell proliferation rate. Thus, the study led to the identification of a suitable hydrogel for osteoblasts.

## 1. Introduction

Tissue engineering is a field that aims to replace, regenerate, or repair damaged tissue [1]. To complete these aims, tissue engineering uses fiber scaffolds [2]; however, these fibrous scaffolds can also be combined with hydrogels to form a composite scaffold [3]. In this study, we chose bioprinting technology to create a composite composed of a fibrous system and a hydrogel. The research aims to demonstrate the functionality of a bioprinting technology that combines hydrogel bioinks containing a cell culture and a 3D cell scaffold. Bioprinting comprises a technology for the transformation of single layers into two- or three-dimensional structures [4]. Moreover, this technology can also be used to print (inject) materials deep into scaffolds or to print material onto scaffolds [5]. Bioprinting technology can, therefore, be employed to create scaffolds, tissues, and organs. Bioprinting technology uses cells as one of the building blocks; cells can be printed together with the culture medium or with biomaterial-based hydrogels, for example, on alginate or hyaluronic acid. Cells comprise the basic structural, functional, and biological units of all living organisms. By combining cells with hydrogels, it is possible to control or develop the properties of the resulting tissue or organ [4,6,7]. One of a number of potential bioprinting options comprises extrusion technology using a computer numeric control (CNC) printer with a three-axis extrusion printhead. Multicellular aggregates are deposited during printing within a bioink-supporting medium according to a computer-generated template and in accordance with the topology of the required biological structure [8,9]. Hydrogels are used as bioinks that provide cells with an environment that features sufficient hydrophilicity, biocompatibility, and mechanical properties. Bioinks exert a favorable effect on cell migration and viability [10]. Bioprinting technology makes use of synthetic hydrogels, e.g., based on polyethylene glycol, and hydrogels fabricated from natural sources, such as hyaluronan [11].

As mentioned above, bioprinting can be used to print materials into or onto a scaffold surface. Several technologies can manufacture scaffolds in tissue engineering. One of the most widely used technologies for the production of scaffolds is electrospinning [12]. Electrospinning technology is used to produce polymeric nanofiber materials from polymer solutions or melts [13]. Nanofibrous materials prepared via electrospinning technology are suitable for tissue engineering in the form of scaffolds due mainly to their structure, which serves to support cell adhesion and proliferation [14,15,16]. However, the disadvantage of these materials concerns their insufficient mechanical properties, which rule out their effective use in combination with hydrogels. The mechanical properties can be enhanced, however, by combining electrospinning with meltblown technology. The meltblown technology is not very used for scaffold formation in tissue engineering, but as we have shown in our study, it is suitable for scaffold formation [17,18]. Meltblown technology produces microfibers with a diameter of 1–10 µm by extruding a polymer melt through a nozzle with holes of a diameter of tenths of a millimeter, followed by intense blowing of hot air [19,20].

The research aims to demonstrate the functionality of a bioprinting technology that combines hydrogel bioinks containing a cell culture and a 3D cell scaffold. Moreover, the research also aims to prove the biocompatibility of the individual components and the beneficial impact of this combination on enhanced cell viability and proliferation and cellular seeding. A biodegradable composite material was selected as a suitable 3D scaffold consisting of a mixture of microfibers and nanofibers [17] at a precisely defined ratio [18]. The scaffold was fabricated in one single step. Commercial hydrogels obtained from a single supplier, characterized by very low viscosity prior to crosslinking (the premise for the efficient penetration of the hydrogels into fine fibrous structures), were used for testing purposes. The hydrogels were based mainly on hyaluronan, which has previously yielded interesting results in combination with human bone osteoblasts [21,22,23].

The results proved the biocompatibility of the injected hydrogel bioinks with the scaffolds. Compared to the scaffold alone, the combination with printed hydrogels resulted in a higher rate of adherent cells immediately following application, enhanced cell viability, and proliferation throughout the structure [24,25,26].

Since the scaffold was developed primarily for the regeneration of bone tissue, one of the objectives of this study was to find a hydrogel that is suitable for its combination with bone osteoblasts. Based on supportive research [27], the maximum cell viability—as expected—was attained employing a combination of hyaluronan hydrogel supplemented with collagen.

## 2. Materials and Methods

### 2.1. Materials

3D micro-nanofibrous Scaffold—Poly-ε-caprolactone (PCL; Mw 45,000; Sigma Aldrich, Taufkirchen, Germany), absolute ethanol, and chloroform (both Penta Chemicals, Czech Republic). Hydrogel HyStem^®^-Basic—pure thiol hyaluronan with thiol-reactive polyethylene glycol crosslinker, and deionized water (ESI.BIO, Alameda, CA, USA). Hydrogel HyStem^®^-C—Thiol hyaluronan with incorporated collagen, thiol-reactive polyethylene glycol crosslinker, and deionized water (ESI.BIO, USA). Hydrogel HyStem^®^-HP—Thiol hyaluronan with incorporated heparin, thiol-reactive polyethylene glycol crosslinker, and deionized water (ESI.BIO, USA). The employed hyaluronan hydrogels are crosslinked within 20 min after the addition of the crosslinker. Hydrogel PEGgel—polyethylene glycol diacrylate with Ciba PEGcure UV photoinitiator, crosslinked by UV radiation (ESI.BIO, USA).

### 2.2. Scaffold Preparation

**Figure 1:**
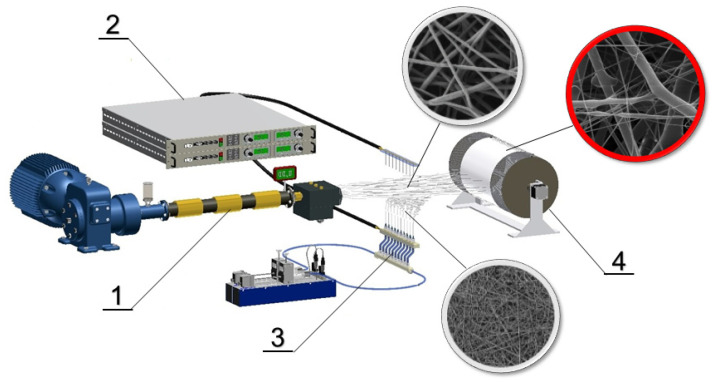
Scheme of a combination of meltblown and electrospinning technology for the production of a 3D scaffold: 1—extruder, 2—high voltage power supply, 3—multi needle spinner, 4—drum collector.

A solution of 16wt% PCL in chloroform/ethanol (9:1) was prepared for the electrospinning process. The scheme of the production equipment set-up is shown in Figure 1. The set-up was composed of a meltblown device (J&M Laboratories, Duluth, GA, USA), an electrospinning device (a multi-needle spinner and a countervailing pressure cylinder), and computer-controlled pumps. The meltblown extruder loading was 100g of polymer per hour. The air velocity was 20 ms^−1^ at 200 mm from a meltblown die. The meltblown die length was 100 mm with 65 orifices with a diameter of 0.4 mm. The needle spinner had 10 needles with a diameter of 1.2 mm with a spacing of 25 mm. The polymer dosage was 70 mL/h. The spinner was charged up to 35 kV positive and the collector 14 kV negative. Fibers were deposited on an intercepting drum collector with a diameter of 350 mm that rotated at 4 rpm.

**Figure 2:**
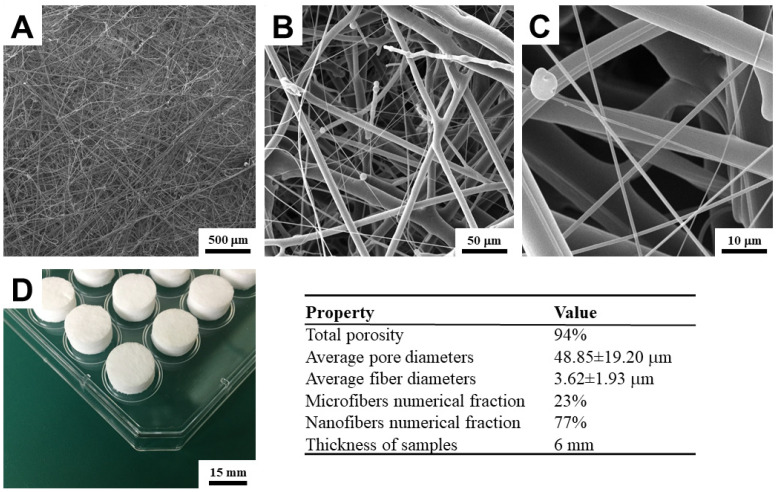
Manufactured 3D scaffolds combining micro- and nanofibers. (**A**–**C**)—scaffold morphology observed with SEM. (**D**)—the image of scaffolds in the form of discs. Table—structural parameters of the material [17].

A single PCL 3D scaffold sheet with a numerical ratio of nanofibers (fiber diameter ≤ 1000 nm) and microfibers (fiber diameter ≥ 1000 nm) of 1:3 was produced for the basic biocompatibility test and subsequent identification of the best hydrogel for bone osteoblasts. The ratio of nanofibers and microfibers was selected from our results from a previous study [17]. The ratio of micro and nanofibers in the scaffold is influenced only by changing the meltblown extruder rotation speed. The layer thickness was 6 mm. The complete structural properties and SEM images of the material are shown in Figure 2.

The gravimetric method was used for the determination of the total porosity. The size of the cut samples of PCL scaffolds was 100 × 100 mm and the thickness was measured using a 49-63 bench micrometer (TMI, New Castle, DE, USA) with a pressure of 400 Pa according to the standard EDANA testing method—NWSP 120.1.R0 (15). The weight and volume of the measured samples in relation to the density of the PLC (1.145 g/cm^3^) were used for the final calculation.

**Figure 3:**
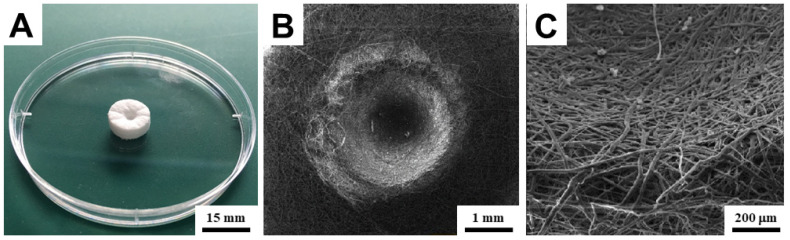
Three-dimensional scaffolds with a pressed trap hole. (**A**)—image of scaffolds with a pressed hole. (**B**)—SEM image of a pressed hole. (**C**)—SEM detail image of the hole wall.

Disks with a diameter of 15 mm were cut out of the fibrous layer (Figure 2). For the first biocompatibility test, holes with a diameter of 3 mm and a depth of 1.5 mm were pressed into the disks (Figure 3). These holes were pressed for a better capture of the surface printing of the bioink drops, particularly to prevent their disintegration immediately after printing and subsequent uneven impregnation into the scaffold structure. Without significantly altering its structural properties, the material permits the insertion of holes (Figure 3). For the second test, comparing the effect of different bioink hydrogels printed directly into the interior of the scaffold, disks of the same size without holes were used. The samples were sterilized with low temperature (37 °C) ethylene oxide for 12 h and subsequently ventilated for three days in a sterile environment. Before bioprinting, they were rinsed three times in phosphate buffer (pH 7.4).

### 2.3. Bioprinting Device

**Figure 4:**
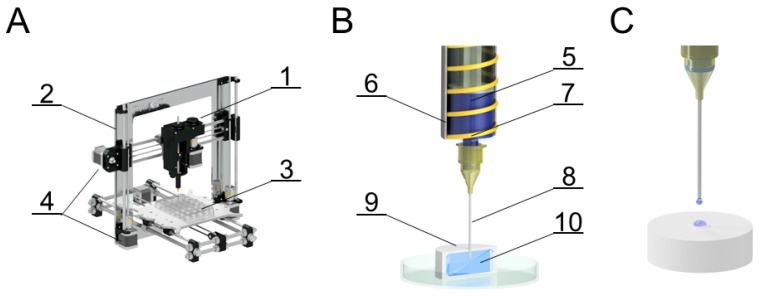
Overall diagram of the bioprinter (**A**); printhead diagram (**B**); printhead detail (**C**). (**A**) 1—printhead, 2—frame, 3—well plate, 4—x and z axis drive, 5—printing medium, 6—case, 7—heating spiral, 8—needle, 9—fiber scaffold sample, 10—extruded medium inside the scaffold.

A three-axis CNC extrusion bioprinter was designed for bioprinting (Figure 4). The printer enables the printhead movement in two x- and z-axes with a precision of 0.1 mm, and a y-axis underlay with a precision of 0.01 mm. The maximum working space is 200 × 200 × 200 mm, the maximum feed rate is 19 mm/s in the x- and y-axes, and 6.4 mm/s in the z-axis. The printhead includes a 2 mL heated print reservoir with a maximum dosing rate of 0.6 mL/s. A minimum theoretical dosing rate is 0.01 µL/s, where for a 0.6 mm diameter needle, the minimum possible pushed drop of the medium is 1.85 µL. A step motor with a 1.8°/step resolution was selected as the printhead piston and individual travels drive. All movements are controlled by the Repetier-Host computer software or by means of a g-code to ensure complete automation of the process. This code can be obtained, for example, from computer-aided design (CAD) software.

Bioprinting was performed in a sterile flowbox with laminar airflow. Prior to printing, all parts of the bioprinter were sterilized with 70% ethanol and UV-C radiation.

### 2.4. Bioink Preparation for Printing

All applied hydrogels had undergone the same preparation process in a sterile environment. After defrosting to ambient temperature, the deionized water (DW) polymer and DW crosslinker were mixed at the 4:1 ratios. A cell pellet formed after centrifugation of the cell suspension and suction of the culture medium was subsequently added to the polymer and DW solution. The pellet contained 9.6 × 10^5^ human bone osteoblasts—MG63 (ATCC, Manassas, VA, USA). Thus prepared cell suspension was stirred for 15 s on a vortex. The cells were after the twelfth passage. The polymer, DW, and cell solution were subsequently mixed with a 1:4 crosslinker and DW solution. Hydrogel bioinks at a concentration of 3.3 × 10^4^ cells/100 µL were thus produced. After mixing the hyaluronan hydrogels solution with the crosslinker solution, spontaneous crosslinking occurred after 20 min. After application, Hydrogel PEGgel was subsequently crosslinked with UV light at 365 nm and 0.13 W/m^2^ for 15 min.

### 2.5. Bioprinting

In the first test, the bioink surface printing consisting of a basic hyaluronan hydrogel (Hydrogel HyStem^®^-Basic) and human osteoblasts (MG63), 3.3 × 10^4^ cells/100 µL was realized. It was a surface print in the form of one 50 µL drop in each hole pressed on a scaffold tablet (HG). Bioink was heated in the reservoir head at 37 °C and was extruded at a rate of 50 µL/s (1.65 × 10^4^ cells per hole). The extruded drop filled the hole in parallel with the surrounding surface of the scaffold within two minutes, and the remainder was evenly charged into the scaffold structure as a regular half-sphere. Twenty minutes after its preparation, the bioink was spontaneously crosslinked after printing. Cells were seeded by conventional pipetting of the standard EMEM medium with 1.65 × 10^4^ cells per hole (SC) on the second set of scaffolds with holes. This set, seeded with cells in a conventional manner, served as a reference.

In the second test, several bioinks were printed in the interior of the scaffolds. Three types of media—Hydrogel HyStem^®^-C, Hydrogel HyStem^®^-HP, Hydrogel PEGgel in admixture with human osteoblasts (MG63), 3.3 × 10^4^ cells/100 µL—were used. A total of 200 µL of bioink were pressed with a needle into the center of each scaffold disk, 3.5 mm below the surface. Bioink heated in the reservoir head at 37 °C and was extruded at a speed of 50 µL/s again. In the case of printing bioink to the structure of the scaffold, 50 µL/s is the maximal value; at an increased speed there was an increased resistance to the extrusion of the hydrogel and its leakage along the needle outside the scaffold observed. During bioink printing, the scaffolds had to be fixed in the culture well by an insert, which prevents the scaffold from lifting along with the needle. After 20 min of their preparation, bioinks with C and HP hydrogels crosslinked their structures spontaneously. After printing, the bioink containing PEG hydrogel was crosslinked for 15 min by means of UV radiation at a wavelength of 365 nm. For comparison, a further set of scaffolds was seeded with cells by conventional surface pipetting of the standard EMEM medium with 6.6 × 10^4^ cells per sample.

The final test analyzed the scaffolds with an internally printed medium in more detail, which obtained the best results in the previous test—Hydrogel HyStem^®^-C. A set of scaffolds seeded with cells by conventional surface pipetting of the classical cell medium was selected as a comparative. Cell counts, bioink volume, and all other variables and methodology were the same as in the previous test.

All cell-seeded materials placed in a 24-well culture plate were subsequently filled by 1.5 mL of culture medium and placed in an incubator. For both tests, a needle of 0.6 mm in diameter and 25 mm in length was used to extrude the medium. The entire bioprinting was carried out in a sterile flowbox at 22 °C and humidity of 55%.

### 2.6. In Vitro Cultivation

Scaffolds with human osteoblasts—MG63 M12 (ATCC, Manassas, VA, USA)—were maintained in eagle’s minimum essential medium (EMEM, Manassas, VA, ATCC, USA) culture medium with 10% FBS (Lonza Biotec, Kourim, Czech Republic) and 1% antibiotics mixture—penicillin/streptomycin/emfotericin B (Lonza Biotec, Kourim, Czech Republic). The cells were cultivated in an incubator (37 °C/5% CO_2_). The medium was changed three times a week—1.5 mL per well.

### 2.7. Bioink Distribution Analysis

The Bioink distribution of the sample structure was analyzed after it was printed into the interior of the structure—200 μL/sample. For the analysis, the computed tomography (CT) method was performed by a microtomograph (SKYSCAN 1272, Bruker, Billerica, MA, USA) and 3D visualization software (SKYSCAN 1.1.9). The sample was shot in x-, y- and z-axis, at 60 kV voltage, 166 µA current, and 648 ms exposure. The scanning rate was 5 µm per frame. Afterwards, the images were reconstructed into a 3D model.

### 2.8. MTT Assay for the Cell Proliferation

Cell viability was monitored after 1, 4, and 7 days using the MTT Assay. For each test day, the MTT Assay of 4 samples was evaluated. Prior to the test, each sample was removed from the well, rinsed with PBS and transferred to another culture plate to avoid distortion of the results by cells that adhered outside the scaffold. Subsequently, 250 µL of MTT solution (2 mg/mL in PBS pH 7.4) was added to 750 μL of medium (EMEM) and incubated together with the defragmented samples (divided into individual laminas) for 3 h at 37 °C and 5% CO_2_. Formazan crystals were dissolved in isopropyl alcohol. After release of all formazan, the sample fragments were thoroughly shaken on the vortex. To remove impurities, the resulting solution was subsequently centrifuged for 2 min at 4000 rpm. The absorption of formazan solution was measured by a spectrophotometer (BioTek EL808, Winooski, VT, USA) at a wavelength of 570 nm (reference wavelength was 650 nm).

### 2.9. Fluorescent Staining

All fluorescence staining of cells in the samples was performed on the 1st, 4th, and 7th day after the scaffolds had been seeded with cells. Prior to initiating the staining protocol, samples had always been rinsed with PBS intensively in order to remove already-dead cells. For the propidium iodide (PI) staining, the cells on the scaffold were fixed in frozen methanol (−20 °C) for 15 min at 4 °C, washed with PBS, and subsequently maintained for 15 min in solution of PI in the dark.

For quantitative cell viability analysis after 1, 4, and 7 days the Live-Dead staining by Calcein AM (Thermo Fisher Scientific) and Ethidium homodimer-1 (EthD-1, Thermo Fisher Scientific) were used. Calcein AM and EthD-1 were diluted in PBS to concentrations of 0.5 μg·mL^−1^ and 2.0 μg·mL^−1^, respectively. The cell-laden scaffold was washed by PBS and subsequently incubated for 30 min at 37 °C in the dark and after that was again washed with PBS.

### 2.10. Fluorescent Microscopy Analysis

Immediately after staining samples, an analysis by means of fluorescence microscopy was carried out. Then the stained samples were rinsed with PBS and observed under a fluorescence microscope (NIKON Eclipse Ti-E, Nikon Imaging, Zbraslav, Czech Republic). The final image creation consisted of composing a picture from images taken with z-axis microscope autofocus at step 1 μm. Snapshot ranges were determined by focusing on the minimum and maximum relief of the surface of the sample. The range and therefore the number of frames were always different for each sample. The surface of the samples was analyzed in the direction of the insertion of bioink—OUTSIDE, and the interior of the samples approximately in the middle of the tablet in the direction of the insertion of bioink—INSIDE. The inside of the sample could be analyzed because the employed micro/nanofibrous scaffolds are made up so that their laminate structure is rigid. After staining the cells with fluorescence, the sample was divided along the mid-lamina into two halves, and then FLM cell migration was possible to be observed 3 mm below the surface.

### 2.11. Cell Count Analysis

Quantification was performed to compare the number of live and dead cells on the test samples. From EthD-1 stained-red fluorescent dead cell nuclei and Calcein AM-green fluorescent-stained live cells (100× magnification), cells from 10 fields of vision were counted and the results were re-counted per cell number per 1 mm^2^. The algorithm enabling image analysis created in Matlab software (MathWorks, Natick, MA, USA) was used to quantify cell nuclei.

## 3. Results and Discussion

**Figure 5:**
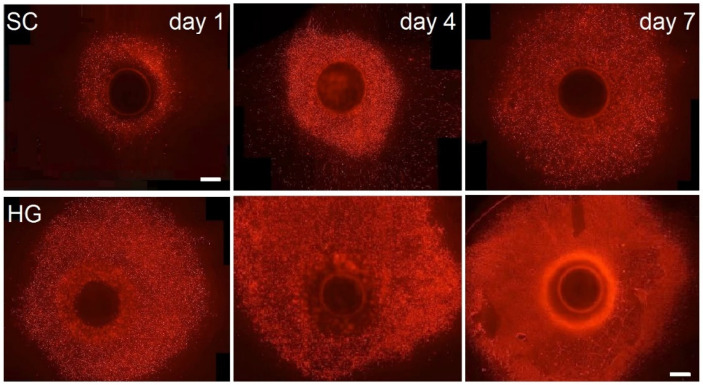
Images from fluorescence microscopy of bone osteoblasts on scaffolds on the 1st, 4th and 7th test days—propidium iodide staining. The images consist of shots taken by microscope auto-focus in z-axis, 1 µm step; the scale is 1 mm. SC—scaffold with conventionally seeded cells, HG—scaffolds with printed hydrogel.

**Figure 6:**
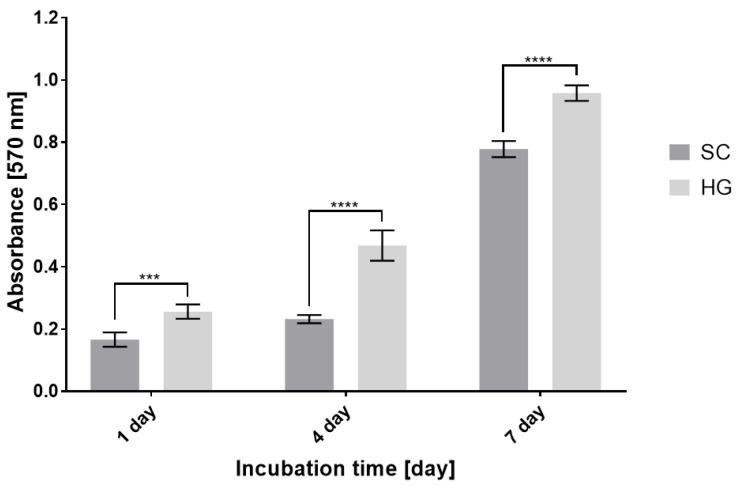
Cell metabolic activity described by the MTT Assay—comparison of surface bioprinting and conventional cell seeding (mean value ± standard deviation, *n* = 4). Statistically significant difference in the significance level: *** *p* < 0.0006; **** *p* < 0.0001. SC—scaffolds with conventionally seeded cells, HG—scaffolds with a printed hydrogel bioink.

The results of in vitro testing show the functionality of the combination of bioprinting with 3D fiber scaffolds and, furthermore, the significant benefits it brings. The combination is fully biocompatible. Fluorescence microscopy (Figure 5) and MTT Assay (Figure 6) representing the basic print-surface test show that scaffolds with seeded cells exhibit a higher degree of cell viability and confluent growth of the scaffold. In addition, in the bioink printed scaffolds, there is a high degree of migration of cells from the point of their printing into the structure of the material. It can be said that hydrogel aids in the initial consolidation and distribution of cells evenly over the structure of the scaffold and prevents the cells from migrating largely in the gravitational-gradient direction. Fluorescence microscopy and MTT Assay values on post-cultivation day one confirm the higher rate of live cells after their seeding, and hence higher efficiency of targeted cell seeding by bioprinting.

**Figure 7:**
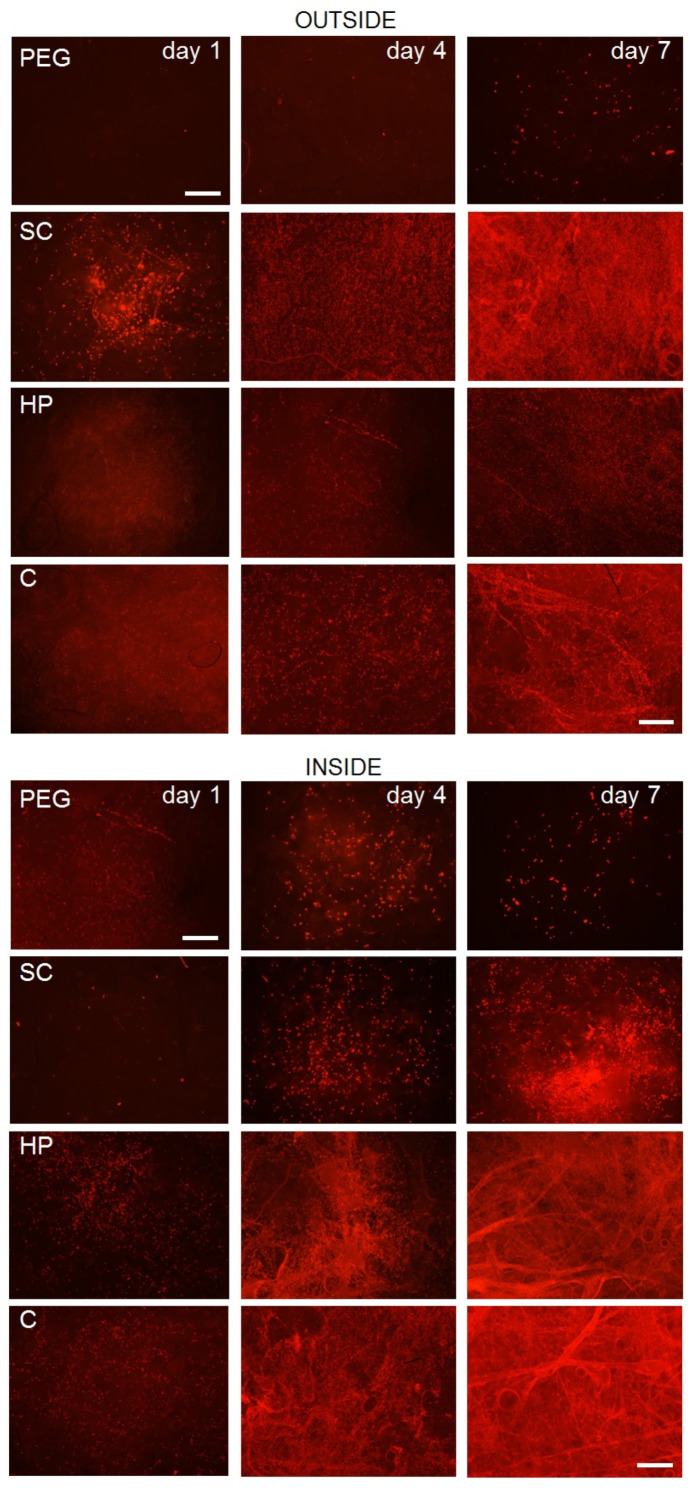
Images of fluorescence microscopy of bone osteoblasts on scaffolds on the 1st, 4th and 7th test days comparing the effect of the internal printing of various bioinks on the viability of human osteoblasts—propidium iodide staining. The images consist of shots taken by microscope auto-focus in z-axis, 1 µm step; the scale is 500 µm. The top section shows surface cell proliferation—OUTSIDE. The bottom part shows internal cell proliferation (3 mm under the surface)—INSIDE. C—Hydrogel with incorporated collagen, HP—Hydrogel with incorporated heparin, PEG—polyethylene glycol photosensitive hydrogel, SC—scaffold with conventionally seeded cells.

**Figure 8:**
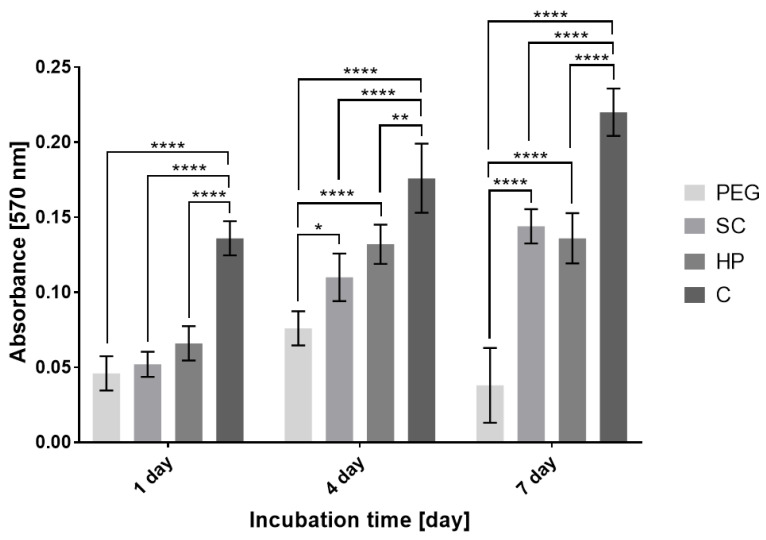
Cell metabolic activity described by the MTT Assay—comparison of the effect of internal bioprinting of different print media on the viability of human osteoblasts (mean value ± standard deviation, n = 4). Statistically significant difference in the significance level: * *p* < 0.04; ** *p* < 0.002; **** *p* < 0.0001 (ANOVA, post-hoc Tukey). C—Hydrogel with incorporated collagen, HP—Hydrogel with incorporated heparin, PEG—polyethylene glycol photosensitive hydrogel. SC—scaffold with conventionally seeded cells.

The results of fluorescence microscopy (Figure 7) and the MTT Assay (Figure 8), which compare the effect of internal bioprinting of various bioinks on the viability of human osteoblasts, show and confirm our assumption that cell viability is best supported by the bioink containing hyaluronan hydrogel with incorporated collagen. This combination, as in the first experiment, demonstrates a higher rate of cell metabolic activity in scaffolds combined with hydrogel. On the other hand, the polyethylene glycol hydrogel has shown a lack of crosslinking by UV radiation, which did not penetrate into the scaffold structure and therefore appeared to be inconvenient. Therefore, it is clear that hydrogels using additional radiation for crosslinking are not suitable for a combination with 3D fiber scaffolds. The results clearly show that unlike conventional cellular seeding, printing cells with hydrogel into the structure will provide a higher degree of internal cell proliferation. The chosen amount (200 µL) of the printed bioink applied to a single sample (15 × 6 mm) has proven to be satisfactory. The sample totally absorbed this volume without creating larger defects or structural changes.

**Figure 9:**
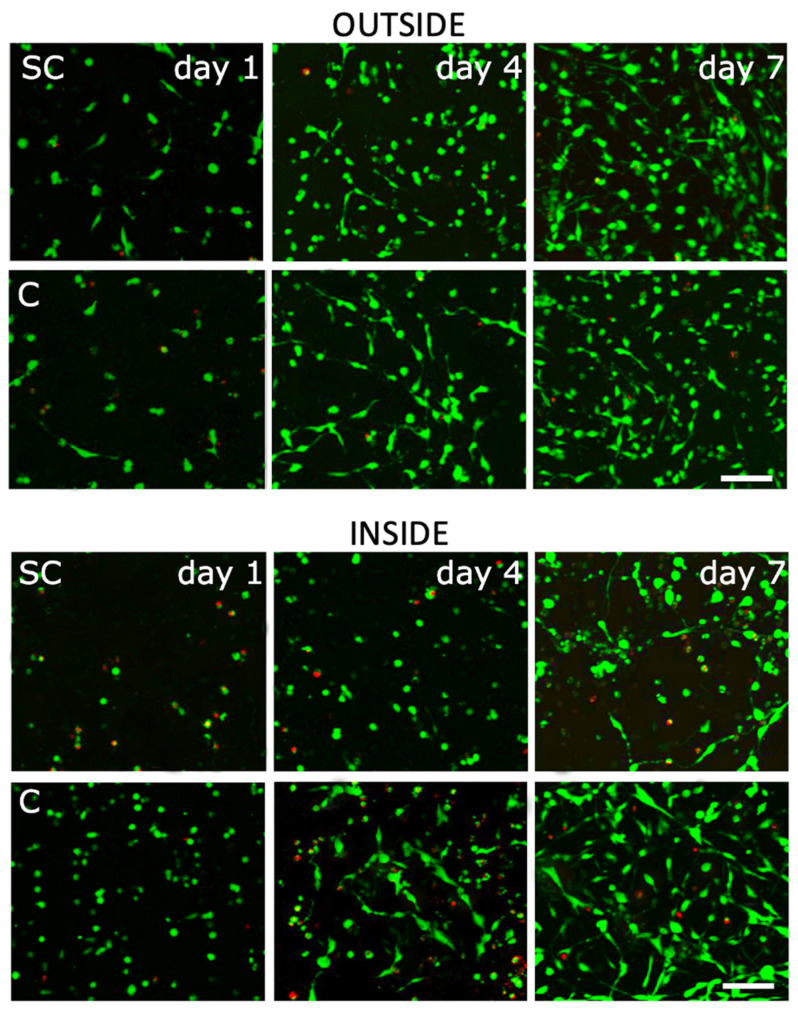
Fluorescence images of live and dead bone osteoblasts on scaffolds of the 1st, 4th and 7th test days comparing the effect of the internal printing of selected bioink and scaffold without bioink on the viability of human osteoblasts—live cells (green: Calcein AM) and dead cells (red: EthD-I) staining. The scale is 100 µm. The top section shows surface cell proliferation—OUTSIDE. The bottom part shows internal cell proliferation (3 mm under the surface)—INSIDE. C—Hydrogel with incorporated collagen, SC—scaffold with conventionally seeded cells.

**Figure 10:**
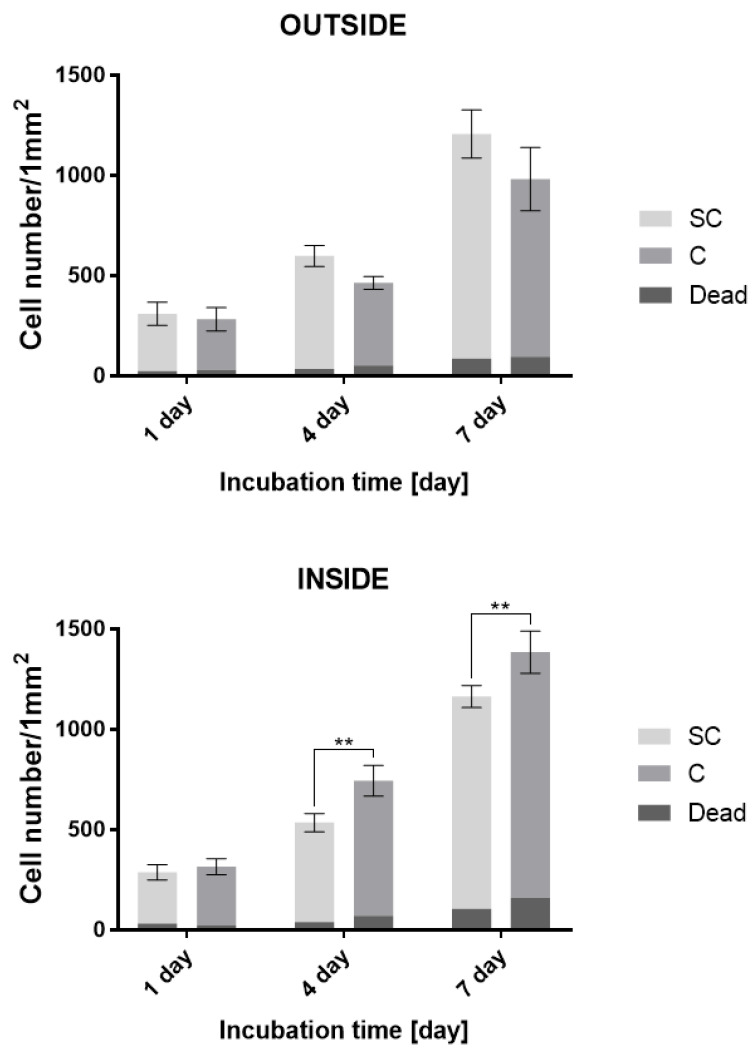
Cell proliferation rate described by cell number on 1 mm^2^—comparison of the effect of internal bioprinting of selected bioink and scaffold without bioink on the viability of human osteoblasts (mean value ± standard deviation, *n* = 10). Statistically significant difference in the significance level: ** *p* < 0.0046 (ANOVA, post-hoc Tuckey). The top section shows the number of cells per mm^2^ on the surface. The bottom part shows the number of cells per mm^2^ into the structure. C—Hydrogel with incorporated collagen, SC—scaffold with conventionally seeded cells, Dead—Number of dead cells.

The final “Live or Dead” test evaluated cell viability, migration, and morphology in more detail with regard to their seeding and the used medium. Analyses using fluorescence microscopy (Figure 9) and the quantification of cellular count (Figure 10) confirmed previous results. The test was primarily aimed at visualizing the cell morphology and distinguishing between the number of red-stained dead and green-stained living cells. The results show that during the seeding of the cells, the printed hydrogel ensures their higher percentage of capture in the scaffold. It can be stated that the hydrogel provides better migration of the cells in the scaffold structure and overall provides a higher viability contrary to a scaffold fitted in a conventional manner without hydrogel; however, this assertion cannot be related to the migration of cells on the surface of the scaffold if we compare the scaffold with the internal printing of selected bioink with the scaffold without bioink, where it is mainly the surface of the scaffold that is seeded. From the overall morphology of cells, it is clear that the hydrogel promotes the distribution and viability of cells, which, however, are less adhered to the surface of the fibers, especially within the structure. It seems that the restriction in cell nutrition in combination with hydrogel inside the scaffold is not great. In the case of a hydrogel-free scaffold, again, a lower level of internal proliferation is evident, but it is also given by the way the cells are seeded. Overall, as shown in Figure 9, in the case of a combination of scaffold and hydrogel, the cells show better morphology, better adherence, better expansion, and a more pronounced shape. This can be observed especially on the fourth and seventh day of incubation. The results of cell proliferation and viability, apparent from fluorescence microscopy, correlate with the analysis of the number of cells per unit area (Figure 10).

The ratio of dead to living cells corresponds to the expected values for this type of application. It can be said that the proportion of dead cells is generally higher inside the scaffold and on the seventh day it reaches 13% in the case of combination with a hydrogel. In the case of both internal and external seeding, the overall proportion of dead cells in combination with the hydrogel is higher than in the fibrous scaffold alone. Nevertheless, the number of viable cells in the case of the combination with the hydrogel is significantly higher in the case of internal seeding on the fourth and seventh day compared to the fiber scaffold alone. The combination of scaffold by hydrogel, therefore, has a significant effect on internal proliferation and cell viability, as confirmed by the analysis of the number of dead and living cells.

**Figure 11:**
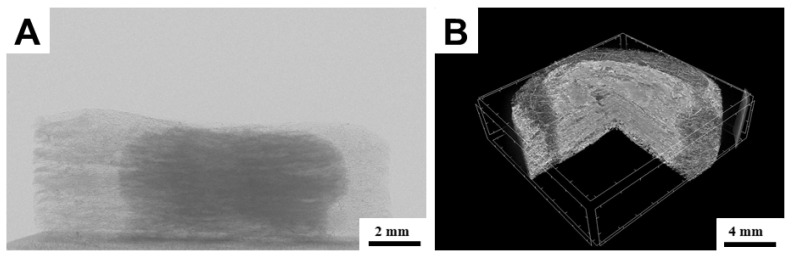
Computed tomography images visualizing the distribution of hydrogel within a scaffold structure. (**A**)-side view of the sample; X-ray scan. (**B**)-3D sample screening.

A numerical tomography method was used to visualize the distribution of hydrogel by the scaffold structure. From the results, it is evident that in the scanning characteristics mentioned in the methodology, the hydrogel component is contrasting and well-distinguishable (Figure 11). It can be seen that the hydrogel printed inside the structure penetrated the scaffold evenly and without any major inhomogeneities. The distribution copies the shape of the scaffold and it can be assumed that gravitation has no noticeable effect on the distribution. In the CT image in part B, there are light segments resembling hydrogel, but they are only imaginary phantoms. All employed hydrogels were tested, and no greater difference in morphology of their distribution was observed. It can be said that the low-viscosity hydrogels are suitable for printing into the inside structure composed of micro/nanofibres before crosslinking.

After critical evaluation of the functionality of the combination of bioprinting hydrogels with 3D micro/nanofibrous scaffolds, it can be concluded that it brings some advantages as well as disadvantages.

**Figure 12:**
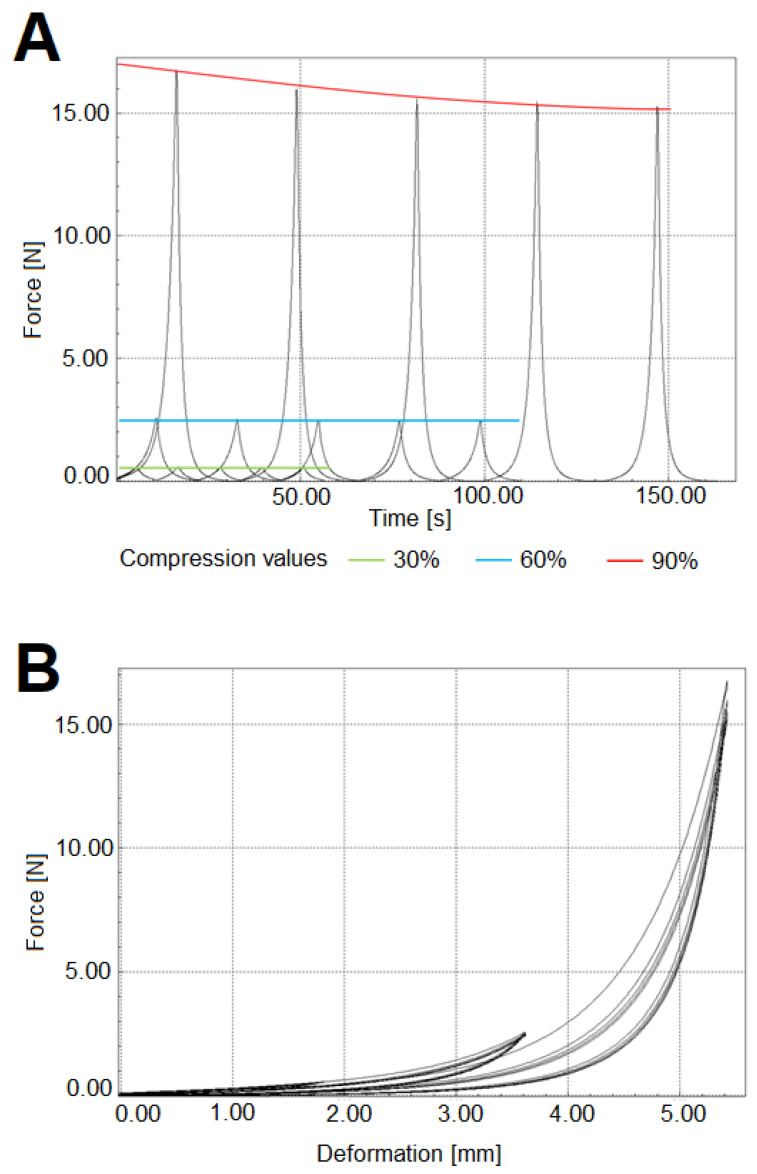
Measurement of cyclic deformation. (**A**) The testing of reversible cyclic deformations of PCL scaffolds at sample compression values 30, 60, and 90%. (**B**) The deformation curves of cyclic deformations testing at sample compression values of 30, 60, and 90%.

The advantages certainly include very good sample handling, where the 3D micro/nanofibrous scaffold is a compact carrier of the hydrogel contained within the structure. A 3D scaffold can be produced as needed in different shapes and sizes. It is also possible to control the density of the fibrous structure and thus significantly adapt the resulting properties of the composite. In the case of PCL, the fibrous structure lends the system a long term degradation component compared to the hydrogel itself. The 3D fibrous scaffold provides the hydrogel system with very good mechanical properties—the sample module is 0.8 MPa and the deflection limit at 60% cyclic compression. The values of the compression modulus and the limited reversible-cyclic deformation was carried out on a two-column universal dynamometer-M2.050 (LaborTech, Czech Republic) with a nominal load capacity of 5 kN. A compression element platform with a diameter of 30 mm and strain gauge with a nominal load of 50 N was used. The compression velocity was 20 mm/min. Five cycles at sample compression values (Discs of 15 mm diameter and 6 mm width) of 30, 60, and 90% were performed. Figure 12 shows graphs of individual measurements.

However, there are some disadvantages too. They include a limited choice of crosslinking principles of hydrogel with respect to the structure of the 3D scaffold, where the use of additional crosslinking principles, such as the supply of energy (e.g., UV) or additional crosslinking with solutions etc., is inappropriate. Furthermore, there is a lower homogeneity of the composite compared to the hydrogel systems themselves and a smaller degree of homogeneity of cell agglomeration compared to hydrogels alone. This results in greater scattering of the results of biological testing. Another undisputed disadvantage is the limitation of biological testing options, especially the limited quality visualization in comparison with transparent hydrogels. It is also problematic to perform “Live or Dead” tests.

The findings of this research will make it possible for further research to carry out more efficient seeding of more extensive fiber scaffolds, as well as scaffolds, which are more complicated in shape. It might be possible to print structured patterns into a 3D fiber structure or patterns of two or more different cell lines or bioinks.

## 4. Conclusions

The experiments served to prove the functionality of a combination of bioprinting technology and 3D fiber scaffolds fabricated from a biodegradable polymer. The scaffolds demonstrated a high level of affinity for the selected bioinks. This combination provided a high level of biocompatibility and evinced an enhanced rate of live cells immediately following seeding, i.e., a beneficial impact on the rate of cell proliferation and confluent growth compared to conventionally-seeded scaffolds. The results thus indicate that a hyaluronan hydrogel with incorporated collagen is suitable for the bioprinting of human osteoblasts within the structure of the scaffold considered in this study. The combination of the selected 3D fiber scaffold and the tested technology appears to be promising in terms of its use in the field of bone regenerative medicine.

## Data Availability

The data presented in this study are available on request from the corresponding author.
